# Prevalence, Associated Factors, and Appropriateness of Empirical Treatment of Trichomoniasis, Bacterial Vaginosis, and Vulvovaginal Candidiasis among Women with Vaginitis

**DOI:** 10.1128/spectrum.00161-23

**Published:** 2023-04-13

**Authors:** Sung-Hsi Huang, Heng-Cheng Hsu, Tai-Fen Lee, Hui-Min Fan, Chi-Wei Tseng, I-Hui Chen, Hung Shen, Chia-Yi Lee, Hui-Ting Tai, Hong-Ming Hsu, Chien-Ching Hung

**Affiliations:** a Department of Internal Medicine, National Taiwan University Hospital Hsin-Chu Branch, Hsinchu, Taiwan; b Department of Tropical Medicine and Parasitology, National Taiwan University College of Medicine, Taipei, Taiwan; c Department of Obstetrics and Gynecology, National Taiwan University Hospital and National Taiwan University College of Medicine, Taipei, Taiwan; d Department of Surgery, National Taiwan University Cancer Center, Taipei, Taiwan; e Graduate Institute of Clinical Medicine, National Taiwan University College of Medicine, Taipei, Taiwan; f Department of Laboratory Medicine, National Taiwan University Hospital and National Taiwan University College of Medicine, Taipei, Taiwan; g Department of Laboratory Medicine, National Taiwan University Hospital Hsin-Chu Branch, Hsinchu, Taiwan; h Department of Obstetrics and Gynecology, National Taiwan University Hospital Hsin-Chu Branch, Hsinchu, Taiwan; i Department of Internal Medicine, National Taiwan University Hospital and National Taiwan University College of Medicine, Taipei, Taiwan; j Department of Internal Medicine, National Taiwan University Hospital Yunlin Branch, Yunlin, Taiwan; Tainan Hospital, Ministry of Health and Welfare

**Keywords:** bacterial vaginosis, diagnostics, empirical treatment, trichomoniasis, vaginitis, vulvovaginal candidiasis

## Abstract

Trichomoniasis (TV), bacterial vaginosis (BV), and vulvovaginal candidiasis (VVC) are the most common causes of vaginitis. This study investigated the prevalence of these diagnoses, their associated factors, and the appropriateness of the empirical treatment. From March 25, 2019, to June 17, 2022, 429 women with symptoms or signs of vaginitis were enrolled in a hospital in northern Taiwan with 438 episodes of vaginitis. Vaginal swabs were collected for Gram’s staining, *in vitro* cultures for Trichomonas vaginalis, bacteria, and yeasts, and multiplex PCR assay for TV, BV, and VVC. Their empirical treatments were recorded. Factors associated with different etiologies of vaginitis were sought in multivariable logistic regression models. The prevalence of TV, BV, and VVC were 2.1%, 22.8%, and 21.7%, respectively, while coinfections of BV and VVC, TV and BV, TV and VVC, and triple infection occurred in 5.0%, 0.2%, 0.2%, and 0.7%, respectively. Multivariable analyses revealed that having multiple sexual partners was associated with TV and BV (adjusted odds ratio [aOR] 9.756 and 3.246, respectively), while menopausal women were less likely to have VVC (aOR 0.184). Moreover, dysuria was associated with TV (aOR 4.981), vaginal itch and pelvic pain with VVC (aOR 3.223 and 0.425, respectively), and discharge pH > 4.5 with BV (aOR 1.767). Other clinical symptoms and pelvic examination features had limited value for differential diagnosis. Among the 78 empirical antifungal and metronidazole prescriptions, 55.2% were ineffective or unnecessary. Our study highlights the importance to integrate appropriate diagnostic tools into the clinical care of women with vaginitis.

**IMPORTANCE** Vaginal complaints are widespread among women and are associated with emotional, physical, and economic burdens with challenges in their diagnosis and management. In this survey, we identified that 40% of vaginitis in Taiwan was caused by either trichomoniasis, bacterial vaginosis, vulvovaginal candidiasis, or a combination of these infections. Our data suggested that typical physical findings appeared infrequently among women with these infections and their empirical treatments were frequently inappropriate. Our findings highlighted the importance of integrating proper diagnostic tools into clinical practice to improve the diagnosis and management of vaginitis, as recommended by national and international guidelines.

## INTRODUCTION

Vaginitis or vaginal complaints are extremely common among women attending primary care or gynecological services (1). Trichomoniasis (TV), bacterial vaginosis (BV), and vulvovaginal candidiasis (VVC) are the most common etiologies of vaginitis worldwide (2, 3). The global prevalence of TV and BV is estimated to be 5.3% and 26%, respectively, with variation among populations with different background characteristics and geographic locations (4, 5). About 75% of women experience VVC at least once in their lifetime (6) and recurrent VVC, defined as four or more episodes of the infection every year, is not uncommon (7). Together, these infections incur significant productivity losses and economic burden (5, 7, 8). Moreover, TV and BV have been found to increase the risk of HIV transmission (9, 10) and adverse pregnancy outcomes (11–13).

It is challenging to differentiate the causes of vaginitis because the symptoms and signs are usually nonspecific (2, 14). Guidelines recommend careful inquiry into the medical and sexual history, physical examination, and use of appropriate laboratory testing, especially point-of-care (POC) diagnostic tools, to determine the etiology before initiation of treatment (1, 15). However, in real-world practices, diagnosis and management of vaginitis remain largely syndromic (16, 17) or empirical (18), without the support of laboratory testing. Even in resource-rich countries, in-office diagnostic tests are not always available and may be frequently underutilized (18–20). The consequences of such practices have been demonstrated in a recent study in the United States, where 42% of empirical prescriptions for women with vaginitis were deemed inappropriate and those who received unnecessary antimicrobials had more return visits for vaginitis (19). This rate of inappropriate empirical treatment was surprisingly high. More studies are needed to confirm the generalizability of such findings.

Despite its common presence, vaginitis attracts disproportionately little attention. In many parts of the world, including most of the Asia-Pacific region, the burden of these infections has yet to be appropriately determined (4, 5, 7, 21). Ineffective or delayed treatment of vaginitis adversely affects women’s physical and mental health (22, 23) and amplified transmission in the cases of TV, while overprescription of treatment may risk increased antimicrobial resistance in the community (24). In recent years, multiplex PCR assays have been used to improve the diagnosis of vaginitis (25, 26). This study aimed to employ a comprehensive array of laboratory tools, including multiplex PCR and *in vitro* culture, to investigate the prevalence of TV, BV, and VVC among women with vaginitis in northern Taiwan, identify factors associated with these diagnoses, and evaluate the appropriateness of empirical treatment.

## RESULTS

From March 25, 2019 to June 17, 2022, 429 women were enrolled with 438 episodes of vaginitis. Nine participants were enrolled for a second time after a median of 519 days after their first enrollment (range, 277 to 1033 days). The median age of the participants was 41.9 years (interquartile range [IQR], 35.8 to 52.0) and the majority were married (71.0%) and had a history of pregnancy (82.6%) (Table 1). Only 4.1% of the participants reported having multiple sexual partners. A history of abnormal vaginal discharge was common (45.2%).

**TABLE 1:** Demographic and medical history of the participants according to their microbiological diagnoses^*a*^

	Total	Trichomoniasis (TV)			Bacterial vaginosis (BV)			Vulvovaginal candidiasis (VVC)		
Demographic and clinical characteristic	(N = 438)	TV (*n* = 9)	No TV (*n* = 429)	*P* value	BV (*n* = 100)	No BV (*n* = 338)	*P* value	VVC (*n* = 95)	No VVC (*n* = 343)	*P* value
Age, yrs	41.9 (35.9, 52.0)	41.9 (36.0, 52.2)	36.3 (34.3, 47.9)	0.171	42.4 (36.2, 52.6)	39.5 (34.2, 49.6)	**0.033**	43.4 (36.3, 53.8)	38.5 (32.6, 44.6)	**<0.001**
Marital status										
Married	311 (71.0%)	5 (55.6%)	306 (71.3%)	0.441	64 (64.0%)	247 (73.1%)	0.148	67 (70.5%)	244 (71.1%)	0.150
Single	85 (19.4%)	3 (33.3%)	82 (19.1%)		26 (26.0%)	59 (17.5%)		23 (24.2%)	62 (18.1%)	
Widower or divorced	42 (9.6%)	1 (11.1%)	41 (9.6%)		10 (10.0%)	32 (9.5%)		5 (5.3%)	37 (10.8%)	
Personal habit										
Smoking	35 (8.0%)	1 (11.1%)	34 (7.9%)	0.531	9 (9.0%)	26 (7.7%)	0.676	7 (7.4%)	28 (8.2%)	>0.999
Alcohol	34 (7.8%)	1 (11.1%)	33 (7.7%)	0.520	7 (7.0%)	27 (8.0%)	0.835	9 (9.5%)	25 (7.3%)	0.516
Douching	18 (4.1%)	1 (11.1%)	17 (4.0%)	0.317	6 (6.0%)	12 (3.6%)	0.263	3 (3.0.2%)	15 (4.4%)	0.774
Multiple sexual partners	18 (4.1%)	2 (22.2%)	16 (3.7%)	**0.048**	9 (9.0%)	9 (2.7%)	**0.009**	2 (2.1%)	16 (4.7%)	0.385
Obstetric history										
Menopaused	122 (27.9%)	3 (33.3%)	119 (27.7%)	0.714	26 (26.0%)	96 (28.4%)	0.704	10 (10.5%)	112 (32.7%)	**<0.001**
History of pregnancy (N = 437)	361 (82.6%)	6 (66.7%)	355 (82.9%)	0.194	79 (79.0%)	282 (83.7%)	0.294	73 (76.8%)	288 (84.2%)	0.125
no. of pregnancy (N = 437)	2 (1, 2)	2 (1, 2)	0 (0, 1)	0.054	2 (1, 2)	2 (0, 2)	0.520	2 (1, 2)	1 (0, 2)	**0.008**
History of abortion (N = 437)	211 (48.3%)	4 (44.4%)	207 (48.4%)	>0.999	50 (50.0%)	161 (47.8%)	0.733	43 (45.3%)	168 (49.1%)	0.562
History of ectopic pregnancy (N = 437)	15 (3.4%)	0 (0%)	15 (3.5%)	>0.999	7 (7.0%)	8 (2.4%)	0.053	4 (4.2%)	11 (3.2%)	0.749
Contraception										
Use of hormonal contraceptives	23 (5.3%)	0 (0%)	23 (5.4%)	>0.999	6 (6.0%)	17 (5.0%)	0.798	6 (6.3%)	17 (5.0%)	0.605
Use of intra-uterine device	15 (3.4%)	1 (11.1%)	14 (3.3%)	0.271	5 (5.0%)	10 (3.0%)	0.349	6 (6.3%)	9 (2.6%)	0.106
Medical history										
Recent use of antibiotics	101 (23.1%)	0 (0%)	101 (23.5%)	0.126	21 (21.0%)	80 (23.7%)	0.685	24 (25.3%)	77 (22.4%)	0.583
History of gynecological cancer	52 (11.9%)	1 (11.1%)	51 (11.9%)	>0.999	14 (14.0%)	38 (11.2%)	0.482	6 (6.3%)	46 (13.4%)	0.072
History of pelvic inflammatory disease	79 (18.0%)	2 (22.2%)	77 (17.9%)	0.668	17 (17.0%)	62 (18.3%)	0.882	20 (21.1%)	59 (17.2%)	0.451
History of vaginal discharge syndrome	198 (45.2%)	7 (77.8%)	191 (44.5%)	0.085	48 (48.0%)	150 (44.4%)	0.568	42 (44.2%)	156 (45.5%)	0.907
History of recurrent urinary tract infection	96 (21.9%)	4 (44.4%)	92 (21.4%)	0.111	24 (24.0%)	72 (21.3%)	0.583	28 (29.5%)	68 (19.8%)	0.050
Symptomatology										
Fever	13 (3.0%)	0 (0%)	13 (3.0%)	>0.999	2 (2.0%)	11 (3.3%)	0.741	2 (2.1%)	11 (3.2%)	0.743
Pelvic pain	94 (21.5%)	2 (22.2%)	92 (21.4%)	>0.999	23 (23.0%)	71 (21.0%)	0.679	13 (13.7%)	81 (23.6%)	**0.047**
Vaginal itch	141 (32.0%)	4 (44.4%)	136 (31.7%)	0.476	26 (26.0%)	114 (33.7%)	0.179	45 (47.4%)	95 (27.7%)	**<0.001**
Vaginal burning sensation	59 (13.5%)	2 (22.2%)	57 (13.3%)	0.347	13 (13.0%)	46 (13.6%)	>0.999	16 (16.8%)	43 (12.5%)	0.308
Vaginal pain	50 (11.4%)	3 (33.3%)	48 (11.1%)	0.075	9 (9.0%)	41 (12.1%)	0.475	12 (12.6%)	38 (11.1%)	0.716
Dysuria	41 (9.3%)	3 (33.3%)	38 (8.9%)	**0.043**	10 (10.0%)	31 (9.2%)	0.845	7 (7.4%)	34 (9.9%)	0.553
Dyspareunia	42 (9.6%)	1 (11.1%)	41 (9.6%)	0.600	12 (12.0%)	30 (8.9%)	0.339	12 (12.6%)	30 (8.7%)	0.244
Concurrent diagnosis										
Concurrent gonorrhea	10 (2.3%)	1 (11.1%)	9 (2.1%)	0.189	4 (4.0%)	6 (1.8%)	0.246	1 (1.1%)	9 (2.6%)	0.697
Current pelvic inflammatory disease	28 (6.4%)	0 (0%)	28 (6.5%)	>0.999	7 (7.0%)	21 (6.2%)	0.816	4 (4.2%)	24 (7.0%)	0.477
Current urinary tract infection	14 (3.2%)	0 (0%)	14 (3.3%)	>0.999	5 (5.0%)	9 (2.7%)	0.327	3 (3.2%)	11 (3.2%)	>0.999
Atrophic vaginitis	19 (4.3%)	0 (0%)	19 (4.4%)	>0.999	5 (5.0%)	14 (4.1%)	0.780	2 (2.1%)	17 (5.0%)	0.391
Concurrent TV	9 (2.1%)	-	–	-	4 (4.0%)	5 (1.5%)	0.125	4 (4.2%)	5 (1.5%)	0.107
Concurrent BV	100 (22.8%)	4 (44.4%)	96 (22.4%)	0.125	-	–	-	25 (26.3%)	75 (21.9%)	0.407
Concurrent VVC	95 (21.7%)	4 (44.4%)	91 (21.2%)	0.107	25 (25.0%)	70 (20.7%)	0.407	-	–	-

a *P*-values in Bold indicate *P*-values < 0.05.

### Prevalence of TV, BV, and VVC.

**FIG 1:**
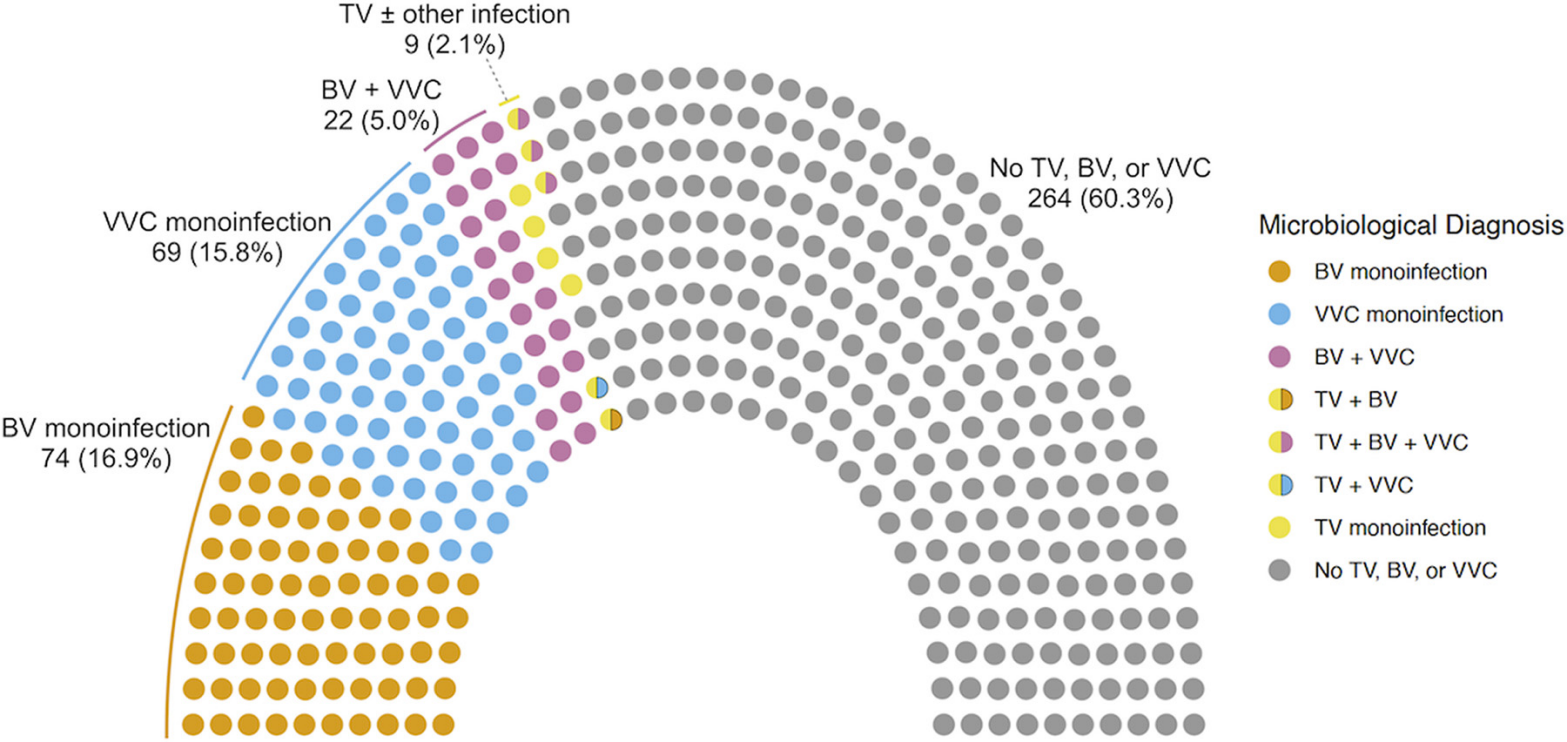
Microbiological diagnosis of vaginitis in a parliament plot.

The prevalence of TV, BV, and VVC were 2.1% (9/438), 22.8% (100/438), and 21.7% (95/438), respectively (Fig. 1). Co-infections occurred in 27 episodes (6.2%), including 22 (5.0%) with BV and VVC, 1 (0.2%) with TV and BV, 1 (0.2%) with TV and VVC, and triple infections in 3 (0.7%). Compared with the composite laboratory diagnosis, *in vitro* TV culture identified 77.8% of cases of TV, Nugent score identified 70.0% of cases of BV, and microscopy and culture identified 85.3% of cases of VVC, while multiplex PCR identified 100%, 54.0%, and 72.6% of TV, BV, and VVC, respectively (Table S1).

### Associated factors with TV, BV, and VVC.

In univariable analysis, having multiple sexual partners and dysuria were associated with TV (*P*-value, 0.048 and 0.043, respectively); age and having multiple sexual partners were associated with BV (*P*-value, 0.033 and 0.009, respectively); and age, menopause, number of pregnancy, pelvic pain, and vaginal itch were associated with VVC (*P*-value, <0.001, <0.001, 0008, 0.047 and < 0.001, respectively) (Table 1).

Typical, textbook pelvic examination findings of TV, BV, and VVC were uncommon among the participants with these confirmed diagnoses (Table 2). Of note, only 28.4% of the participants with TV had frothy or purulent vaginal discharge and only 50.0% had a pH > 4.5. Similarly, fishy or odorous vaginal discharge (14.8%) and a pH > 4.5 (39.0%), which represented signature findings of BV, appeared in a minority of the participants with BV. Moreover, only 7.1% of VVC patient manifested with thick, cheesy discharge.

**TABLE 2:** Findings in pelvic examination according to the microbiological diagnoses^*a*^

		Trichomoniasis (TV)			Bacterial vaginosis (BV)			Vulvovaginal candidiasis (VVC)		
Finding of pelvic exam	Overall	TV (*n* = 9)	No TV (*n* = 429)	*P* value	BV (*n* = 100)	No BV (*n* = 338)	*P* value	VVC (*n* = 95)	No VVC (*n* = 343)	*P* value
Discharge amt (N = 366)										
Trace	105 (28.7%)	3 (33.3%)	102 (28.6%)	0.145	25 (30.1%)	80 (28.3%)	0.977	22 (26.2%)	83 (29.3%)	**0.001**
Small	100 (27.3%)	2 (22.2%)	98 (27.5%)		23 (27.7%)	77 (27.2%)		12 (14.5%)	88 (31.1%)	
Moderate	121 (33.1%)	1 (11.1%)	120 (33.6%)		27 (32.5%)	94 (33.2%)		33 (39.8%)	88 (31.1%)	
Large	40 (10.9%)	3 (33.3%)	37 (10.4%)		8 (9.6%)	32 (11.3%)		16 (19.3%)	24 (8.5%)	
Discharge color (N = 380)										
Clear	104 (27.4%)	2 (22.2%)	102 (27.5%)	0.966	27 (30.7%)	77 (26.4%)	0.643	20 (23.5%)	84 (28.5%)	0.198
White	167 (43.9%)	5 (55.6%)	162 (43.7%)		40 (45.5%)	127 (43.5%)		46 (54.1%)	121 (41.0%)	
Yellow	66 (17.4%)	1 (11.1%)	65 (17.5%)		14 (15.9%)	52 (17.8%)		11 (12.9%)	55 (18.6%)	
Bloody	43 (11.3%)	1 (11.1%)	42 (11.3%)		7 (8.0%)	36 (12.3%)		8 (9.4%)	35 (11.9%)	
Discharge consistency (N = 378)										
Cheesy	16 (4.2%)	0 (0%)	16 (4.3%)	0.882	4 (4.5%)	12 (4.1%)	0.435	6 (7.1%)	10 (3.4%)	0.140
Frothy	106 (28.0%)	2 (22.2%)	104 (28.2%)		25 (28.4%)	81 (27.9%)		29 (34.1%)	77 (26.3%)	
Mucoid	94 (24.9%)	3 (33.3%)	91 (24.7%)		27 (30.7%)	67 (23.1%)		21 (24.7%)	73 (24.9%)	
Watery	162 (42.9%)	4 (44.4%)	158 (42.8%)		32 (36.4%)	130 (44.8%)		29 (34.1%)	133 (45.4%)	
FoμL or odorous discharge (N = 384)	40 (10.4%)	1 (11.1%)	39 (10.4%)	>0.999	13 (14.8%)	27 (9.1%)	0.162	9 (10.5%)	31 (10.4%)	>0.999
pH > 4.5 (N = 339)	99 (29.2%)	4 (50.0%)	95 (28.7%)	0.238	30 (39.0%)	69 (26.3%)	**0.045**	21 (27.3%)	78 (29.8%)	0.776
With cervicitis (N = 432)	17 (3.9%)	1 (11.1%)	16 (3.8%)	0.306	5 (5.1%)	12 (3.6%)	0.556	2 (2.1%)	15 (4.4%)	0.547

a *P*-values in Bold indicate *P*-values < 0.05.

In multivariable analysis, having multiple sexual partners was independently associated with a higher risk of TV (adjust odds ratio [aOR] 9.756, 95% CI 1.639–58.088) and BV (aOR 3.246, 95% CI 1.005 to 10.189), while menopause was associated with lower risk of VVC (aOR 0.184, 95% CI 0.077 to 0.390) (Table 3). A few symptoms and signs were found to be associated with specific diagnosis: presenting with dysuria was associated with TV (aOR 4.981, 95% CI 1.102 to 22.506), vaginal itch with VVC (aOR 3.223, 95% CI 1.888 to 5.559), pelvic pain with VVC (aOR 0.425, 95% CI 0.204 to 0.829), and a vaginal discharge pH > 4.5 with BV (aOR 1.767, 95% CI 1.023 to 3.028). Other clinical histories, symptoms, or physical findings were not associated with these diagnoses, including the use of hormonal contraceptives, intrauterine devices, or antibiotics, and the amount, color, consistency, or smell of the vaginal fluid. The findings were generally similar in different models in the sensitivity analysis (Table S2 and Table S3).

**TABLE 3:** Associated factors with trichomoniasis, bacterial vaginosis, and vulvovaginal candidiasis in multivariable analyses^*a*^

	Trichomoniasis		Bacterial vaginosis		Vulvovaginal candidiasis	
Variable in the model	Adjusted odds ratio	*P* value	Adjusted odds ratio	*P* value	Adjusted odds ratio	*P* value
Menopaused	-	–	-	–	0.184 (0.077–0.390)	**<0.001**
History of ectopic pregnancy	-	–	3.053 (0.847–10.553)	0.075	-	–
Multiple sexual partners	9.756 (1.639–58.088)	**0.012**	3.246 (1.005–10.189)	**0.042**	-	–
History of vaginal discharge	4.214 (0.831–21.374)	0.083	-	–	-	–
History of gynecological cancer	-	–	-	–	0.471 (0.153–1.201)	0.145
Vaginal itch	-	–	-	–	3.223 (1.888–5.559)	**<0.001**
Pelvic pain	-	–	-	–	0.425 (0.204–0.829)	**0.016**
Dysuria	4.981 (1.102–22.506)	**0.037**	-	–	-	–
Vaginal discharge pH > 4.5	-	–	1.767 (1.023–3.028)	**0.039**	-	–

a *P*-values in Bold indicate *P*-values < 0.05.

### Appropriateness of empirical treatment.

**FIG 2:**
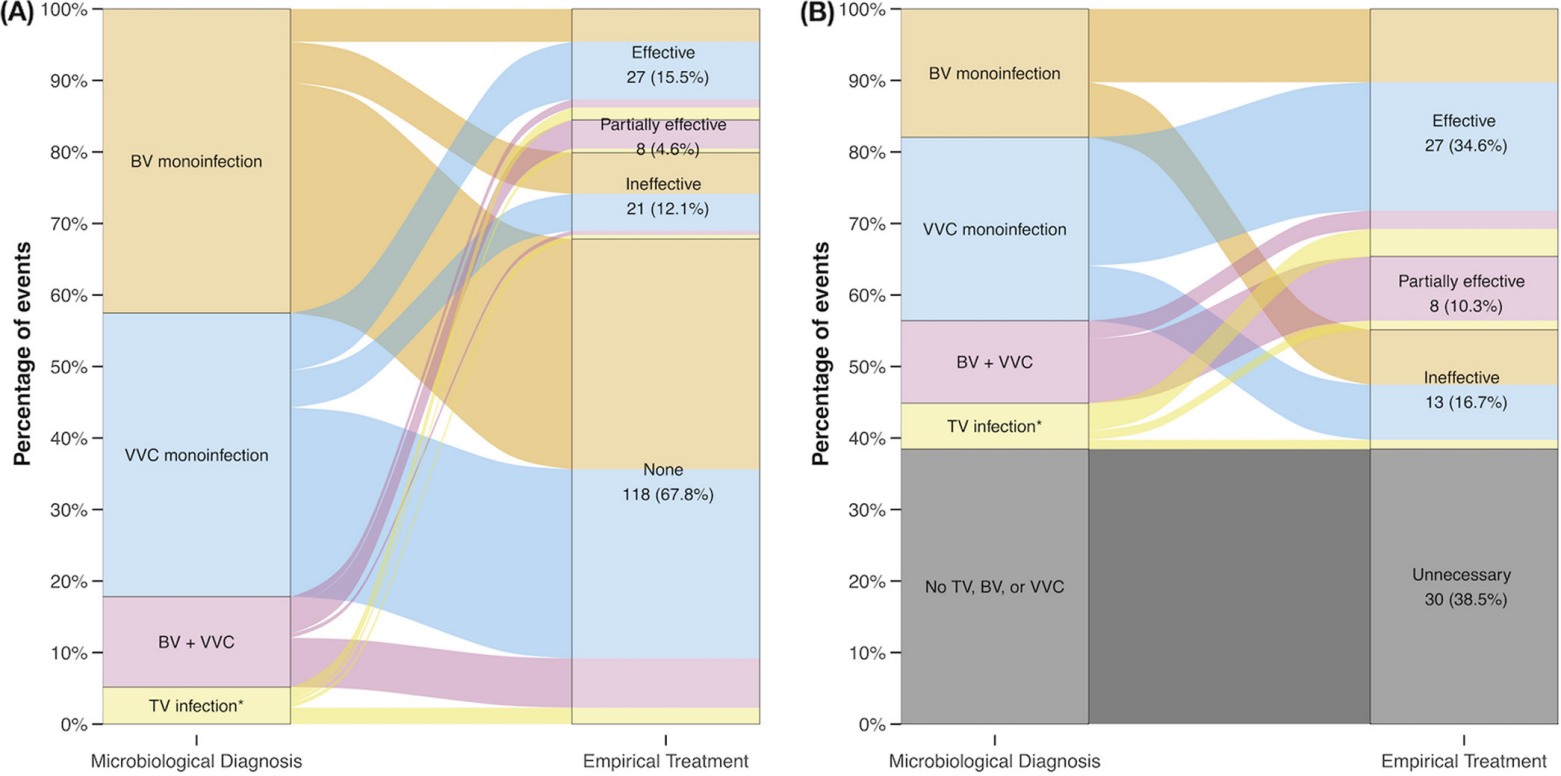
Alluvial diagram illustrating the mismatch between microbiological diagnosis and effectiveness of empirical antimicrobial treatment among (A) events with confirmed microbiological diagnosis (*n* = 174), and (B) events with empirical treatment containing antifungal agents and/or metronidazole (*n* = 78). BV, bacterial vaginosis; VVC, vulvovaginal candidiasis; TV, trichomoniasis. Asterisks indicate trichomoniasis with or without other coinfections.

Ninety-nine (22.6%) episodes of vaginitis were treated empirically with one or more systemic or topical antimicrobial agents, including antifungals in 53 prescriptions, metronidazole in 35 prescriptions and others (Table 4 and Fig. S1). Patients with recurrent or more pronounced vaginal symptoms were more likely to receive empirical antimicrobial treatment (Table S4). Of the 174 events with confirmed TV, BV, or VVC, 20.1% were empirically treated with effective or partially effective antimicrobial agents, while 12.1% were treated with ineffective agents (Fig. 2A). Among 9 participants with confirmed TV, 5 (55.5%) were treated empirically and 4 (80%) were effective. In 100 episodes of BV, 30 (30.0%) were treated empirically and 14 (46.7%) were effective. In 95 episodes of VVC, 34 (35.8%) were treated empirically and 20 (58.8%) were effective.

**TABLE 4:** Empirical antimicrobial regimens prescribed to women with specific microbiological diagnosis^*a*^

Antimicrobial agent	TV monoinfection (N = 4)	BV monoinfection (N = 74)	VVC monoinfection (N = 69)	TV+BV (N = 1)	TV+VVC (N = 1)	BV+VVC (N = 22)	TV+BV+VVC (N = 3)	No TV, BV, or VVC (N = 264)	Total (*n* =438)
Antifungal agents	3	8	14	1	0	6	0	21	53
Metronidazole	3	8	8	0	0	5	1	10	35
Third generation cephalosporin	0	2	5	0	0	0	0	4	11
Macrolides or doxycycline	0	4	7	0	0	1	0	8	20
Other beta-lactams	0	2	2	0	0	1	0	9	14
No empirical treatment	1	56	46	0	1	12	2	221	339

a BV, bacterial vaginosis; TV, trichomoniasis; VVC, vulvovaginal candidiasis. Both topical and systemic antimicrobials were included (Because patients could receive more than 1 empirical antimicrobial treatment, the total number of treatment could be higher than the number of cases in each column.).

Among 78 prescriptions containing empirical metronidazole and/or antifungal agents, 34.6% were effective, 10.3% partially effective, 16.7% ineffective, and 38.5% unnecessary (Fig. 2B). A similar result was also observed in sensitivity analysis, after excluding the episodes with gonorrhea, pelvic inflammatory disease, or urinary tract infection (Fig. S2).

## DISCUSSION

Through comprehensive laboratory investigations, we identified that BV and VVC were similarly common among women with vaginitis in northern Taiwan, each accounting for more than one-fifth of episodes of vaginitis, while TV occurred in every one out of 48. Typical symptoms and physical findings of TV, BV, or VVC were observed in a minority of the participants with these infections and the empirical treatment was frequently ineffective, unnecessary, or delayed.

TV continued to be the most common nonviral sexually transmitted infections worldwide with an increased global incidence between 1999 and 2019 (27). This study identified a low but unneglectable presence of TV with a prevalence of 2.1%, consistent with the estimated regional prevalence of 2.5% in the South-East Asia region (4) and close to the prevalence of 2.2% in another study from northern Taiwan (28). The prevalence of BV in this study (22.8%) was also similar to the global and regional prevalence (26% and 24%, respectively). The prevalence of VVC was slightly lower than that in the previous reports (32% to 45.3%) (19, 29), and this could be attributed to the older age of our participants because VVC is more prevalent in women of reproductive age (6, 30). Estrogen could generate an immunotolerant vaginal microenvironment, thereby facilitating fungal overgrowth and contributing to the pathogenesis of VVC (31).

Several clinical factors were found to be independently associated with TV, BV, or VVC. Of note, having multiple sexual partners was identified as the strongest associated factor with TV and BV, supporting the importance of sexual history in the diagnosis and management of vaginitis. We also found that dysuria occurred more frequently in women with TV. As shown in previous reports, TV could be an important, but often ignored, differential diagnosis in patients with dysuria or recurrent urinary tract infection (32–34). For the diagnosis of VVC, the integration of the three independent associated factors (menstruation status, vaginal itch, and pelvic pain) provided pretest probabilities that could be clinically useful (Table S5). For instance, in premenopausal women with vaginal itch and no pelvic pain, the proportion with confirmed VVC was almost 50%, while in menopausal women with pelvic pain but not vaginal itch, the proportion was 0%. Further validation will be needed to confirm the generalizability of these predictors.

Because symptoms may be subjective to patients’ perception, many clinicians rely heavily on the physical findings to reach a tentative diagnosis and determine treatment. However, as shown in this study, none of the physical features were significantly associated with any microbiological diagnoses of vaginitis, probably because the clinical severity of these infections varied among the participants. The high rate of coinfections could also complicate the clinical pictures (19, 35). Consequently, prescribing empirical antimicrobial treatment for women with vaginitis could be extremely challenging. As shown in this study, less than 50% of antimicrobial treatment prescribed empirically was effective or partially effective. In community practice settings, Hillier et al. also showed that, among women with a laboratory-confirmed etiologic diagnosis of vaginitis, approximately half of the prescriptions were inappropriate (19). Both over- and underprescription of antimicrobials could adversely influence the individuals and the community, as mentioned in many studies (5, 10–13). Taking these considerations together, we strongly suggest that POC or other laboratory tests shall be incorporated into clinicians’ practices to improve diagnosis and care for women with vaginal complaints, as recommended by international or national guidelines (1, 14, 15).

The study utilized multiplex PCR as part of the laboratory investigation for vaginitis, revealing some diagnostic benefits. In contrast to other diagnostic methods, TV, BV, and VVC could be simultaneously detected in one multiplex PCR assay. Moreover, traditional methods, such as *in vitro* TV culture and reading of Nugent score for BV and Gram stain for VVC, are usually more labor-intensive, with interpretation more subjective to experience (36) and accuracy occasionally questioned (37). Multiplex PCR serves as a sensitive and objective platform for investigating the etiology of vaginitis in several studies in Europe and in the United States (26, 38, 39). Nevertheless, the application of this method in the Asian population is yet to be validated. In this study, multiplex PCR outperformed traditional methods in diagnosing TV but had lower detection rates for BV and VVC (Table S1). The percent agreement between the results from Nugent score and multiplex PCR in this study was also suboptimal (83.0%, Cohen’s Kappa = 0.302). Because vaginal microbiota could vary according to ethnicity and environmental factors (36), how to optimize multiplex PCR assay for diagnosis of BV among Asian populations warrants further investigation.

Our study was performed with a few limitations. First, despite thorough laboratory investigations for TV, BV, and VVC, more than 50% of the participants with vaginitis did not have a microbiological diagnosis, indicating that other etiologies might be involved. In this study, 4.3% and 2.3% of participants had atrophic vaginitis and gonorrhea, respectively. Other causes, including aerobic vaginitis (14, 40), chlamydia and other sexually transmitted infections, and other noninfectious causes, were not investigated in this study. Therefore, we were unable to provide more insights into these important issues. Also, the small number of confirmed TV infection makes evaluation of the appropriateness of their empirical treatment difficult and these numbers should be interpreted with caution. Second, we did not use selective culture media to isolate *Candida* species. However, the plates of blood agar and chocolate agar used herein both supported the growth of *Candida* species (41, 42), and these culture media identified 65.3% of the participants with VVC, showing sensitivity only slightly less than PCR assay (detection rate, 72.6%). With the integration of microscopic and molecular diagnoses, we believe that the prevalence of VVC found in this study remains a valid estimate. Third, to understand the appropriateness of the empirical treatment for vaginitis, the gynecologists were encouraged to manage their patients as per their routine practices. However, it would be understandable that some of them might have chosen to wait for the laboratory results before initiation of treatment, especially in cases of participants with milder symptoms (Table S4). This could explain the low rate of empirical antimicrobial therapy in this study.

In summary, TV, BV, and VVC were found in 40% of women with vaginitis in our local survey. The varied clinical presentations make the diagnosis without proper testing difficult and empirical treatment of vaginitis challenging. Integration of proper diagnostic tools into clinical practice is encouraged to improve the care of these women.

## MATERIALS AND METHODS

### Study procedure and participant enrollment.

The study was conducted at the outpatient gynecologic clinic in National Taiwan University Hospital, Hsin-Chu Branch (NTUH-HC), a regional teaching hospital in northern Taiwan that provided both primary and tertiary health care services. Women at least 20 years of age were prospectively enrolled if they presented with symptoms or signs of vaginitis, including abnormal or increased vaginal discharge, vaginal itch, vaginal pain, vaginal burning sensation, or dyspareunia. Pregnant women were excluded from the study. After giving informed consent, participants provided detailed medical history and underwent pelvic examination by the attending gynecologists, when vaginal pH was determined and vaginal swabs were collected for research investigations. Before and during the research period, other POC tests, including whiff test, on-site microscopic examination, and rapid antigen tests, were either unavailable or unutilized in this hospital. Empirical treatment of vaginitis was provided by the attending gynecologists according to their clinical judgment. Participants were allowed to be enrolled for a second time if they had recurrent symptoms or signs of vaginitis at least 6 months after the initial enrollment.

The study was approved by the Research Ethics Committee in NTUH-HC (registration number, 107-088-E) and was carried out according to the principles expressed in the Declaration of Helsinki.

### Outcomes and definitions.

This study used both traditional diagnostic methods and multiplex PCR for diagnosis of TV, BV, and VVC. Traditional diagnostic methods included *in vitro* culture for TV, Nugent score for BV, and Gram stain and culture for VVC (1, 25). Microbiological diagnosis of TV was confirmed either by observing motile flagellated trichomonads in culture medium suspension or by detecting DNA extracts with PCR assay (43). BV was defined by a Nugent score of 7 or greater or positive multiplex PCR assay (44). VVC was defined by the isolation of *Candida* species in aerobic culture, identification of yeast-like pathogens and their pseudohyphae in Gram stain, or positive PCR of either Candida glabrata, C. krusei, or other *Candida* spp. (39). “Coinfection” was defined by microbiological detection for more than one infection among TV, BV, and VVC. While gonorrhea was not the primary target of the study, diagnosis of gonorrhea was made if Gram-negative diplococci were found in the smear or if Neisseria gonorrhoeae was isolated from aerobic cultures.

Empirical antimicrobial treatment, including both systemic and topical agents, was categorized into “effective,” “partially effective,” “ineffective,” “unnecessary,” or “no treatment.” The treatment was deemed “effective” against TV, BV, or VVC if it contained the treatment regimen(s) recommended by the World Health Organization or by the Centers for Diseases Control and Prevention (1, 15). For coinfections, empirical treatment was considered “partially effective” if it contained antimicrobial agents against at least one, but not all, coexisting pathogens. Empirical treatment that did not fulfill the above criteria was deemed “ineffective” if the prescribed antimicrobial did not match a microbiological diagnosis, or “unnecessary” if treatment was prescribed to an event with no microbiological diagnosis. Those who did not receive empirical treatment were categorized into the “no treatment” group.

### Laboratory investigations.

Vaginal fluid pH value was determined by commercialized pH test paper (Universal pH test paper, ADVANTECT, Japan) during the pelvic examination. A Gram stain smear was examined by trained technicians in the laboratory to determine the Nugent score and the presence of Gram-negative diplococci and yeasts.

Immediately after collection, one vaginal swab (sterile cotton swab) was inoculated in TYI medium containing 1,000 U penicillin, 1,000 μg/mL streptomycin, 2.5 μM amphotericin B, and 0.1% agar for the cultivation of T. vaginalis and kept in a 37°C incubator for daily observation of the presence of motile trichomonads (45, 46). One vaginal sample was collected with an ESwab (Copan Diagnostics Inc, CA, USA) and inoculated into different culture media (Trypticase soy agar with 5% sheep blood, MacConkey agar, thioglycolate medium, anaerobic blood agar plate, chocolate agar, and CDC anaerobic phenylethyl alcohol agar) within 2 h of collection for common aerobic and anaerobic pathogen detection. The isolated colonies were identified by matrix-assisted laser desorption ionization-time of flight (MALDI-TOF). Another ESwab was stored at −20°C until being tested for multiplex PCR for TV, BV, and VVC (BD MAX Vaginal Panel, Becton, Dickinson and Company, USA) (19).

All the above laboratory investigations were individually processed in 3 laboratories: one for all Gram staining and cultures of bacteria and fungi, one for cultures of T. vaginalis, and the other for multiplex PCR assay. Technicians in this study were unaware of the clinical diagnoses, empirical treatment, or test results from the other 2 laboratories.

### Statistical analysis.

The characteristics of the enrolled participants were summarized using median and interquartile range for continuous variables, while categorical variables were described using frequency and percentage. Continuous variables were compared between events with and without specific microbiological diagnoses using Wilcox test, while categorical variables were compared using Fisher’s exact test. To identify independent factors associated with TV, BV, and VVC, variables with *P*-values of < 0.1 in the univariable analysis were entered into multivariable logistic regression models with stepwise backward selection. The models were adjusted for coinfections and missing values were treated by exclusion. Sensitivity analyses were carried out using (1) the data set that included only the first enrollment of each participant, and (2) the data set that excluded those with concurrent urinary tract infection or pelvic inflammatory disease. The statistical analyses were performed using the R statistics software (version 4.2.0). A *P*-value of < 0.05 was considered statistically significant throughout the analyses.

### Data availability.

Deidentified participant-level data will be available upon the publication of the study. Requests for data should be sent to the corresponding author.
