# Supplementary material for: Prevalence, Associated Factors, and Appropriateness of Empirical Treatment of Trichomoniasis, Bacterial Vaginosis, and Vulvovaginal Candidiasis among Women with Vaginitis
Source: Microbiol Spectr. 2023 Apr 13;11(3):e00161-23. doi: 10.1128/spectrum.00161-23 (PMC10269550; doi:10.1128/spectrum.00161-23)
Supplement: Supplemental file 1 — Supplemental material. Download spectrum.00161-23-s0001.pdf, PDF file, 0.3 MB [file spectrum.00161-23-s0001.pdf]

Supplementary Figure S1. Alluvial diagram illustrating the relationship between microbiological diagnosis and effectiveness of empirical antimicrobial treatment. (Abbreviations: BV, bacterial vaginosis; VVC, vulvovaginal candidiasis; TV, trichomoniasis) (Footnote: Asterisk marks indicate the specified infections with or without other co-infections)

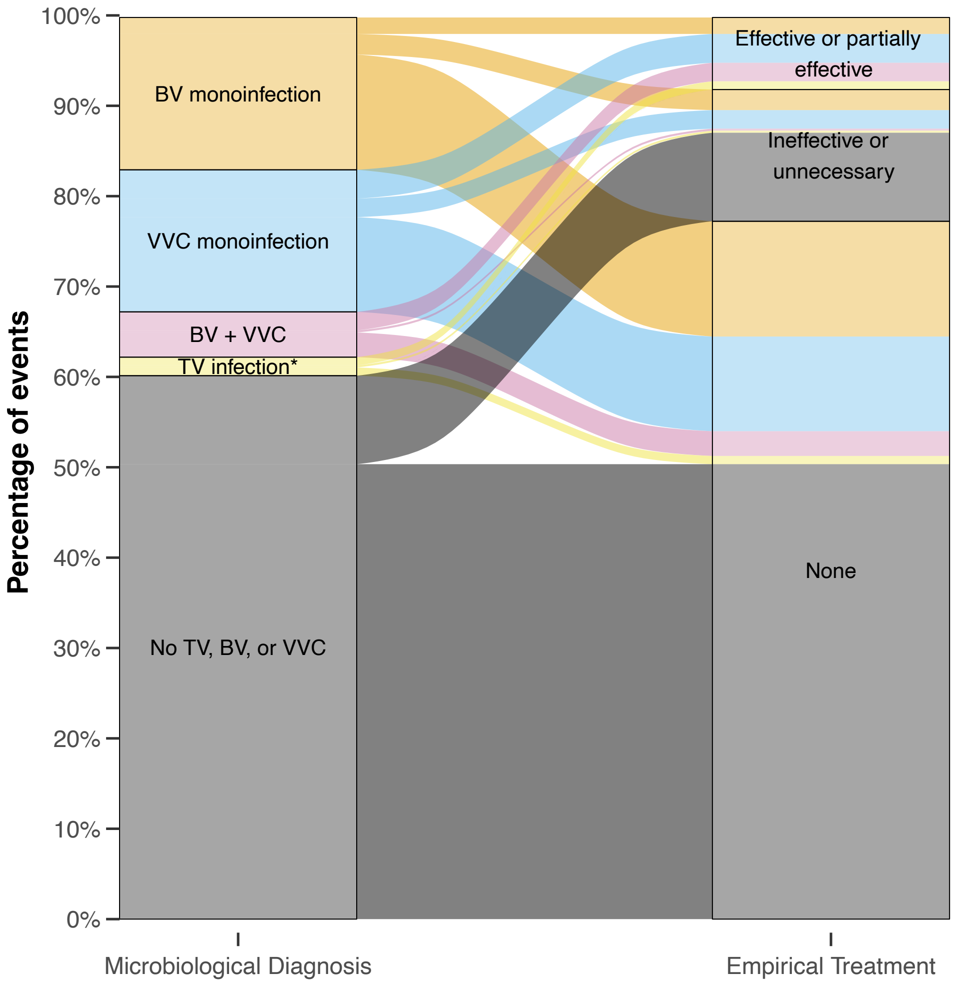

Supplementary Figure S2. Microbiological diagnosis and effectiveness of empirical antimicrobial treatment in (A) events with confirmed microbiological diagnosis (n = 152), and (B) events with empirical antifungal or metronidazole treatment (n = 60) after excluding events with gonorrhea, pelvic inflammatory disease, or urinary tract infection. (Abbreviations: BV, bacterial vaginosis; VVC, vulvovaginal candidiasis; TV, trichomoniasis) (Footnote: Asterisk marks indicate trichomoniasis with or without other co-infections)

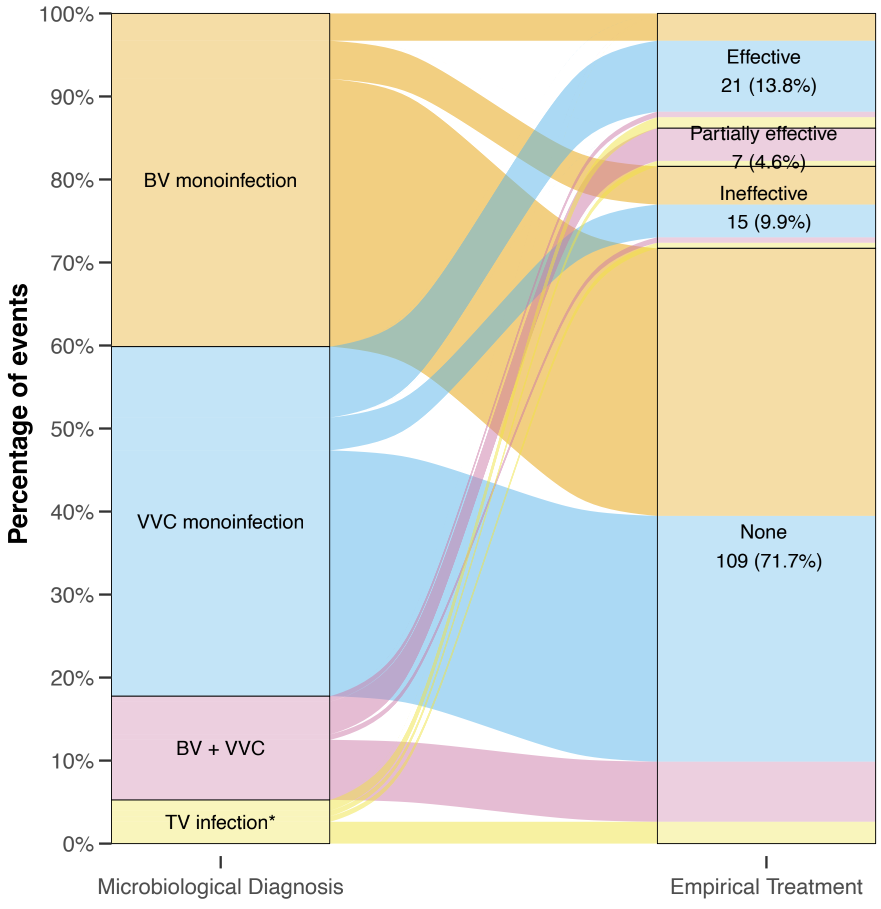

(A)

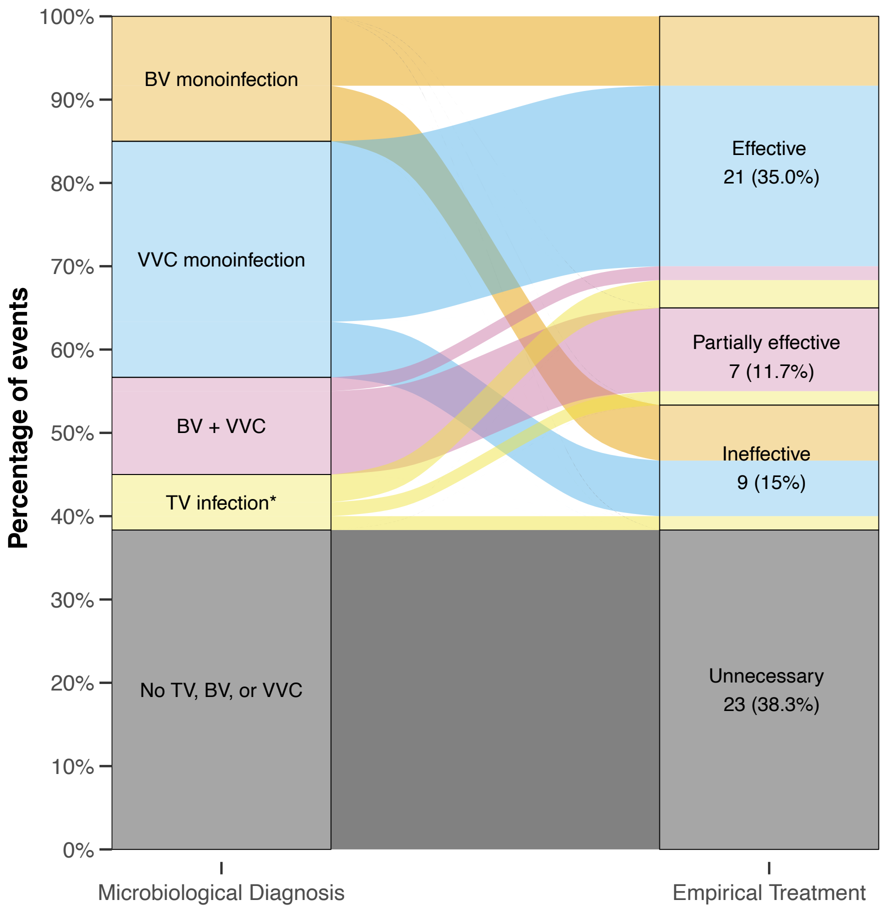

Percentage of events
100%
90%
80%
70%
60%
50%
40%
30%
20%
10%
0%
BV monoinfection
VVC monoinfection
BV + VVC
TV infection*
No TV, BV, or VVC
Effective
21 (35.0%)
Partially effective
7 (11.7%)
Ineffective
9 (15%)
Unnecessary
23 (38.3%)
Microbiological Diagnosis
Empirical Treatment

(B)

Supplement Table S1. Agreement between traditional and molecular diagnostic methods for (A) trichomoniasis, (B) bacterial vaginosis, and (C) vulvovaginal candidiasis. (Tests with invalid multiplex PCR were excluded from the calculation of Cohen's Kappa.)

(A)

	TV culture negative	TV culture positive	Total
Multiplex PCR negative	420	0	420
Multiplex PCR positive	2	7	9
Multiplex PCR invalid	9	0	9
Total	429	7	438
Cohen's Kappa = 0.873 (95% C.I. = 0.698 -1.000)			

(B)

	Nugent score			
	0-3	4-6	7-10	Total
Multiplex PCR negative	183	144	42	369
Multiplex PCR positive	14	16	24	54
Multiplex PCR invalid	9	2	4	15
Total	206	162	70	438
Cohen's Kappa (Nugent ≥ 7 and positive PCR) = 0.302 (95% C.I. = 0.180 – 0.424)				

(C)

	Microscopy and culture both negative	Microscopy positive and/or culture positive	Total
Multiplex PCR negative	338	22	360
Multiplex PCR positive	14	55	69
Multiplex PCR invalid	5	4	9
Total	357	81	438
Cohen's Kappa = 0.703 (95% C.I. = 0.612 – 0.794)			

Supplement Table S2. Associated factors with trichomoniasis, bacterial vaginosis, and vulvovaginal candidiasis in multivariable analyses with dataset excluding the second enrollment among the participants who were enrolled twice.

	Trichomoniasis		Bacterial vaginosis		Vulvovaginal candidiasis	
	Adjusted odds ratio	*p*-value	Adjusted odds ratio	*p*-value	Adjusted odds ratio	*p*-value
Age	--	--	--	--	0.972 (0.937 – 1.008)	0.128
Menopaused	--	--	--	--	0.311 (0.100 – 0.875)	**0.033**
History of ectopic pregnancy	--	--	3.096 (0.905 – 10.596)	0.072	--	--
Multiple sexual partners	13.622 (2.052 – 90.444)	**0.007**	3.989 (1.231 – 12.924)	**0.021**	--	--
History of vaginal discharge	3.904 (0.722 – 21.101)	0.113	--	--	--	--
History of recurrent urinary tract infection	--	--	--	--	1.797 (0.947 – 3.375)	0.070
History of gynecological cancer	--	--	--	--	0.481 (0.154 – 1.248)	0.163
Vaginal itch	--	--	--	--	3.375 (1.922 – 5.999)	**<0.001**
Pelvic pain	--	--	--	--	0.367 (0.157 – 0.783)	**0.014**
Dysuria	5.471 (1.164 – 25.701)	**0.031**	--	--	--	--
Vaginal discharge pH > 4.5	--	--	1.800 (1.038 – 3.122)	**0.036**	--	--

Supplement Table S3. Associated factors with trichomoniasis, bacterial vaginosis, and vulvovaginal candidiasis in multivariable analyses after exclusion of the participants with concurrent pelvic inflammatory disease and urinary tract infection

	Trichomoniasis		Bacterial vaginosis		Vulvovaginal candidiasis	
	Adjusted odds ratio	*p*-value	Adjusted odds ratio	*p*-value	Adjusted odds ratio	*p*-value
Menopaused	--	--	--	--	0.137 (0.056 – 0.338)	**<0.001**
Multiple sexual partners	12.466 (1.863 – 83.419)	**0.009**	4.686 (1.347 – 17.006)	**0.014**	--	--
History of vaginal discharge	5.225 (0.999 – 27.329)	0.050	--	--	--	--
History of gynecological cancer	--	--	--	--	0.400 (0.132 – 1.214)	0.106
Vaginal itch	--	--	--	--	3.128 (1.801 – 5.432)	**<0.001**
Pelvic pain	--	--	--	--	0.387 (0.190 – 0.791)	**0.009**
Dysuria	5.219 (1.143 – 23.840)	**0.033**	--	--	--	--
Vaginal discharge pH > 4.5	--	--	2.071 (1.158 – 3.679)	**0.013**	--	--

Supplement Table S4. Factors associated with prescription of empirical antimicrobial treatment among women with vaginitis.

	Total	With empirical treatment	No empirical treatment	Univariable *p*-value	Multivariable *p*-value
Age, years	41.9 (35.9, 52.0)	42.9 (36.1, 53.2)	39.2 (35.4, 47.7)	**0.008**	0.071
Marital status				0.329	
Married	311 (71%)	66 (66.7%)	245 (72.3%)		
Single	85 (19.4%)	20 (20.2%)	65 (19.2%)		
Widower or divorced	42 (9.6%)	13 (13.1%)	29 (8.6%)		
Smoking	35 (8%)	11 (11.1%)	24 (7.1%)	0.207	
Alcohol	34 (7.8%)	12 (12.1%)	22 (6.5%)	0.085	
Douching	18 (4.1%)	7 (7.1%)	11 (3.2%)	0.144	
Multiple_partner	18 (4.1%)	5 (5.1%)	13 (3.8%)	0.571	
History of pregnancy (N = 437)	361 (82.6%)	80 (80.8%)	281 (83.1%)	0.651	
Oral contraceptives	23 (5.3%)	6 (6.1%)	17 (5%)	0.618	
Intrauterine devices	15 (3.4%)	9 (9.1%)	6 (1.8%)	**0.002**	**<0.001**
Recent antibiotics	101 (23.1%)	35 (35.4%)	66 (19.5%)	**0.002**	
Gynecological malignancy	52 (11.9%)	9 (9.1%)	43 (12.7%)	0.381	
History of pelvic inflammatory disease	79 (18%)	28 (28.3%)	51 (15%)	**0.004**	
History of vaginal discharge syndrome	198 (45.2%)	65 (65.7%)	133 (39.2%)	**<0.001**	**0.036**
History of recurrent urinary tract infection	96 (21.9%)	30 (30.3%)	66 (19.5%)	**0.027**	
Fever	13 (3%)	6 (6.1%)	7 (2.1%)	0.083	
Pelvic pain	94 (21.5%)	27 (27.3%)	67 (19.8%)	0.126	
Vaginal itch	140 (32%)	51 (51.5%)	89 (26.3%)	**<0.001**	**0.028**
Vaginal burning	59 (13.5%)	29 (29.3%)	30 (8.8%)	**<0.001**	**0.003**
Vaginal pain	50 (11.4%)	20 (20.2%)	30 (8.8%)	**0.004**	
Dysuria	41 (9.4%)	20 (20.2%)	21 (6.2%)	**<0.001**	
Dyspareunia	42 (9.6%)	16 (16.2%)	26 (7.7%)	**0.018**	

Large amount of discharge (N = 366)	161 (44%)	52 (57.8%)	109 (39.5%)	**0.003**	0.099
Discharge color (N = 380)				0.116	
Clear	104 (27.4%)	18 (19.6%)	86 (29.9%)		
White	167 (43.9%)	41 (44.6%)	126 (43.8%)		
Yellow	66 (17.4%)	22 (23.9%)	44 (15.3%)		
Bloody	43 (11.3%)	11 (12%)	32 (11.1%)		
Discharge consistency (N = 378)				**0.001**	
Mucoid	94 (24.9%)	15 (16.3%)	79 (27.6%)		Ref
Watery	162 (42.9%)	37 (40.2%)	125 (43.7%)		0.087
Cheesy	16 (4.2%)	11 (12%)	5 (1.7%)		**<0.001**
Frothy	106 (28%)	29 (31.5%)	77 (26.9%)		0.052
Odorous smell (N = 384)	40 (10.4%)	8 (8.5%)	32 (11%)	0.564	
Vaginal pH > 4.5 (N = 339)	99 (29.2%)	28 (34.6%)	71 (27.5%)	0.262	
Cervicitis (N = 432)	17 (3.9%)	4 (4.1%)	13 (3.9%)	>0.999	

Supplement Table S5. Proportion of participants with microbiologically confirmed VVC according to their menstruation status, presence of vaginal itch, and presence of pelvic pain.

Menstruation status	Vaginal itch	Pelvic pain	Probability, % (n/N)
Premenopausal	Presence	Absence	49.3 (33/67)
Premenopausal	Absence	Absence	22.9 (40/175)
Premenopausal	Presence	Presence	21.4 (6/28)
Premenopausal	Absence	Presence	13.0 (6/46)
Menopausal	Presence	Absence	14.3 (5/35)
Menopausal	Absence	Absence	6.0 (4/67)
Menopausal	Presence	Presence	10.0 (1/10)
Menopausal	Absence	Presence	0 (0/10)
